# Waist Circumference Measured by Bioelectrical Impedance Analysis Is Interchangeable with Manual Measurement: Increased Waist Circumference Is Associated with Locomotive Syndrome Risk

**DOI:** 10.1155/2019/5971030

**Published:** 2019-09-25

**Authors:** Satoshi Tanaka, Kei Ando, Kazuyoshi Kobayashi, Taisuke Seki, Shinya Ishizuka, Masaaki Machino, Masayoshi Morozumi, Shunsuke Kanbara, Sadayuki Ito, Taro Inoue, Naoki Ishiguro, Yukiharu Hasegawa, Shiro Imagama

**Affiliations:** ^1^Department of Orthopaedic Surgery, Nagoya University Graduate School of Medicine, Nagoya, Aichi, Japan; ^2^Department of Rehabilitation, Kansai University of Welfare Science, Osaka, Japan

## Abstract

**Objectives:**

The importance of preventive medicine in an aging society is increasing. Locomotive syndrome (LS) is attracting increasing attention. Recently, advances in bioelectrical impedance analysis (BIA) devices have made it possible to automatically measure waist circumference (WC). Nevertheless, there have been no reports evaluating the agreement or interchangeability between WC measured manually and using BIA. Therefore, we aimed to perform these analyses in the context of health checkups and investigated the associations with LS risk.

**Methods:**

We enrolled 597 participants who underwent the following: two-step tests and stand-up tests; evaluations using a 25-question geriatric locomotive function scale for the LS risk test; anthropometric marker measurements including WC measured by manual and BIA; and measurements of total cholesterol and triglycerides. We used Bland–Altman analysis to calculate agreement and interchangeability of the WC measurement using BIA and the manual method. A statistical comparative study was then conducted between normal and LS risk groups. Subsequently, significant risk factors for LS were investigated using multivariate analysis.

**Results:**

The Bland–Altman analysis showed that bias (BIA-manual) was negative overall (−2.024), for males (−1.418) and for females (−2.460), suggesting underestimation using BIA compared with manual measurements. Interchangeability was found between WC measurement by BIA and by manual methods, because the percentage error was less than 15% overall (12.3%), for males (10.2%) and for females (13.8%). Univariate analysis showed that WC was significantly higher in the LS risk group than in the normal group. Multivariate analysis adjusted for confounding factors showed that increase in WC significantly correlated with LS risk.

**Conclusions:**

BIA and manual methods for measuring WC are interchangeable. The increase in WC measured by BIA was significantly associated with LS risk. It is important to continue focusing on increased WC and early detection of LS risk.

## 1. Introduction

In Japan, the average life expectancy has increased in parallel with advanced medical advances. The importance of preventive medicine is implied by phenomena such as prolonged hospitalization due to decreased mobility in elderly people.

In 2007, the Japanese Orthopaedic Association (JOA) proposed the concept of “locomotive syndrome” (LS). LS is a condition in high-risk individuals with musculoskeletal disease who are likely to require nursing care at some point [1]. In 2013, the JOA proposed the following tests to estimate the risk of LS: the two-step test; the stand-up test; and a 25-question geriatric locomotive function scale (GLFS-25). On the basis of these test results, mobility and stage of LS can be determined. LS risk levels are categorized into two stages: 1 and 2. Risk level 1 represents the population in whom movement function has started to decline and for whom measures to prevent deterioration to LS should be instituted [2, 3]. Early diagnosis of LS risk level is important to permit treatment of these conditions, and investigation of the relationships with LS risk from a broad perspective is necessary [4, 5].

Previously, we reported that waist circumference (WC) was significantly associated with LS in elderly females, and this association was more significant than that for other obesity-related parameters [6]. However, in that report, LS risk was not evaluated; moreover, males were not included.

Body composition analysis using bioelectrical impedance analysis (BIA) easily measures water content and body fat mass; it is commonly performed in the context of general medical examinations [7–9]. In recent years, the evolution of BIA devices has been remarkable such that devices can automatically measure not only WC but also the circumference of the neck, chest, and hip in a short period of time. Nevertheless, there have been no reports validating WC by BIA and manual methods, particularly in terms of interrater reliability, agreement, and interchangeability.

Therefore, the purpose of this study was to validate WC measurement by BIA and by manual methods. For this purpose, Spearman correlation and interclass correlation coefficient (ICC) as well as Bland–Altman analysis [10] was used to examine whether these two measurements are interchangeable. Then, we examined whether WC measurement by BIA correlates with LS risk according to sex in a large-scale prospective general health checkup population.

## 2. Materials and Methods

### 2.1. Participants

The study participants were volunteers who underwent health checkups supported at the local government of the town of Yakumo, Japan, in 2016-2017. Yakumo town has a population of approximately 17,000 people, of which 28% are over 65 years old. More people are engaged in agriculture and fishery than those in urban areas. Checkups have been conducted annually in this town since 1982. The checkup consists of voluntary orthopedic and physical function examinations, internal medical examinations, and psychological tests, as well as a health-related quality of life (QoL) survey [11–14]. In this study, participants underwent LS risk tests, anthropometric marker measurements including WC measured manually and by BIA, and blood test. The BIA measurement conditions such as consumption of food and beverage were based on previous report [15] as much as possible, so they underwent these evaluations on an empty stomach. These tests and measurements were performed in the order of blood test, BIA, and LS risk tests. Exclusion criteria were as follows: history of spine and limb joint surgery; severe knee injury; severe osteoarthritis; history of fracture in the hip and spine; neurological disorders; severe mental illness; diabetes; kidney or heart disease; not empty stomach; and severe disability in walking or standing or any dysfunction of the central or peripheral nervous systems.

Among the 1094 individuals who underwent the health checkups, 597 participants (250 males and 347 females) met the selection criteria.

The study protocol was approved by the ethics committee of human research and the institutional review board of our university (No. 2014-0207). All participants provided written informed consent prior to participation. The study procedures were carried out in accordance with the principles of the Declaration of Helsinki.

### 2.2. GLFS-25

The GLFS-25 is a self-administered questionnaire consisting of 25 items graded on a 5-point scale, from no impairment (0 points) to severe impairment (4 points) [16, 17]. The sum of the 25-item scores yields a total possible score between 0 and 100, with increasing values indicating increasing severity of LS. The validity and reliability of this instrument have been reported to be satisfactory, with LS defined by a score ≥16 points and non-LS as ≤15 points. For this study, we used “Locomo 25,” the Japanese version of the GLFS-25.

### 2.3. Two-Step Test

The two-step test measures stride length to evaluate walking ability, including muscle strength, balance, and flexibility of the lower limbs [3]. Subjects stood with the toes of both feet behind a starting line and were then instructed to take two long steps (as long as possible) and then align both feet. The length of the two steps from the starting line to the tips of the toes at the point where the subject stopped was measured. The two-step test score was calculated as the length of the two steps (cm) divided by height (cm).

### 2.4. Stand-Up Test

In the stand-up test, leg strength was assessed by having the subject stand up on one or both legs from a specified height. The subject stood up from each of four seats of heights (40, 30, 20, and 10 cm) in descending height order, first with both legs then with one leg. A subject who could stand up without leaning back to gain momentum and could maintain the posture for 3 s was considered to have passed that height level [3]. In this study, a subject who was unable to stand up on one leg (right or left) from a height of 40 cm was considered to have failed the test.

### 2.5. LS Risk Test

The JOA defines two stages of LS risk. LS risk stage 1 is defined as a two-step test score <1.3, difficulty with one-leg standing from a 40 cm seat in the stand-up test (either leg), or a GLFS-25 score ≥7. Subjects meeting any of these criteria were diagnosed as starting to experience a decline in mobility. LS risk stage 2 is defined as a two-step test score <1.1, difficulty with standing from a 20 cm seat using both legs in the stand-up test, or a GLFS-25 score ≥16. Subjects meeting any of these criteria were diagnosed as having progression of decline in mobility. In this study, subjects who met the criteria for LS risk test stage 1 or 2 were defined as LS risk subjects and the other subjects were defined as normal [4, 5].

### 2.6. Anthropometric Measurements

Anthropometric data including weight, body mass index (BMI), percent body fat (PBF), WC, and appendicular skeletal muscle mass (aSMI) as muscle mass were measured using BIA. The Inbody 770 BIA unit (Inbody Co., Ltd., Seoul, Korea), used to differentiate tissues (such as fat, muscle, and bone) based on their electrical impedance, was utilized [7–9, 18–20]. The accuracy of this device has been reported by various approaches [21, 22]. Individuals grasped the handles of the analyzer, in which electrodes are embedded, and stood on the platform, with the sole of the feet in contact with the electrodes (two electrodes for each foot and hand). BMI was calculated using the following formula: weight (kg)/height^2^ (m^2^). WC by BIA was calculated automatically using the Inbody 770 BIA device. The aSMI was calculated using the following formula: aSMI = arm and leg skeletal muscle mass (kg)/height^2^ (m^2^) [23]. WC was measured manually at the level of the umbilicus at the end of gentle expiration with the subject standing using a nonstretchable measuring tape and recorded in centimeters to the nearest millimeter [6].

### 2.7. Blood Test

We obtained venous blood samples to measure total cholesterol and triglycerides (related to metabolic syndrome). Biochemical analyses of the blood samples were performed using an autoanalyzer (JCA-RX20; Nihon Denshi, Tokyo, Japan).

### 2.8. Statistical Analysis

Continuous variables were expressed as means and standard deviations (SDs), while categorical variables were expressed as percentages. The Kolmogorov–Smirnov test was used to evaluate the normality of the distribution of the data, so the Mann–Whitney *U* test and Fisher's exact test were used to evaluate between-group differences, as appropriate for the data distribution. The correlations between manual methods and BIA for measuring WC were examined using the Spearman *r* and ICC (absolute agreement, two-way random, and single measures). The following cutoff values were used to interpret Spearman correlations: *r* < 0.20 = very weak; 0.20 to 0.39 = weak; 0.40 to 0.59 = moderate; 0.60 to 0.79 = strong; and 0.80 to 1.0 = very strong [23]. The cutoff values to interpret the ICC were as follows: <0.20 = slight; 0.20 to 0.39 = fair; 0.40 to 0.59 = moderate; 0.60 to 0.79 = substantial; and 0.80 to 1.0 = almost perfect [24]. To examine the level of agreement between the manual and BIA measurements, Bland–Altman analysis was used [10]. The mean of the difference between measurements (BIA-manual) was defined as bias, and standard deviation (SD) was also used to calculate 95% confidence limits of agreement (LOA, bias ± 1.96 SD). Bland–Altman plots graphically display the mean of the two measured values (WC measurement using BIA and the manual method) on the *X*-axis and the difference (BIA-manual) between measured values on the *Y*-axis. Whether BIA is interchangeable with the manual method was defined using percentage error (the ratio of 1.96 SD to the mean value of the manual) as 15% or less [25]. To determine the factors associated with LS risk among the variables that exhibited differences (*P* < 0.25) in the univariate analyses, logistic regression analysis using a stepwise method was performed using the aforementioned variables as covariates. A *P* value of <0.05 was considered significant in all analyses. Statistical analyses were conducted using JMP Pro version 13.1 for Mac (SAS Institute Inc., Cary, NC, USA).

## 3. Results

The average age of the 579 participants was 64.6 years (range, 40–88; SD: 10.1 years), the average BMI was 23.5 kg/m^2^, and the average PBF was 29.1%.

Table 1 shows the demographic, anthropometric, blood test, and LS risk prevalence data. There were significant differences between males and females for all variables.

**Figure 1:**
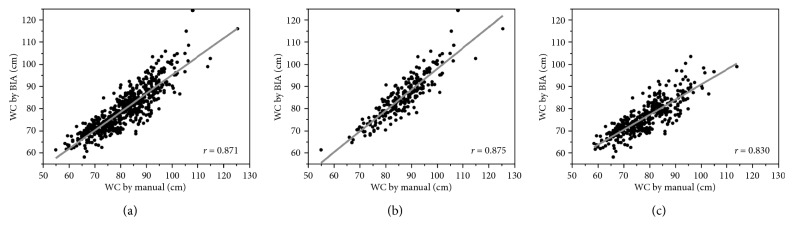
Scatter plot of WC by manual method and BIA. WC measured by BIA had significantly very strong positive correlation with that of the manual method. (a) Total (*r* = 871, *P* < 0.001). (b) Male (*r* = 0.875, *P* < 0.001). (c) Female (*r* = 0.830, *P* < 0.001).

**Figure 2:**
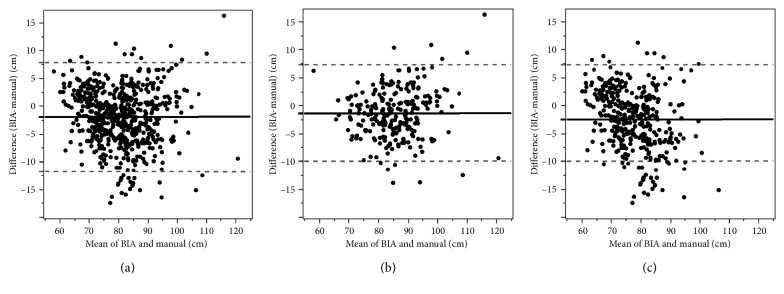
Bland–Altman plot of difference in WC (BIA measurement minus manual measurement) against the mean of two measurements. The middle line denotes bias (mean difference between the two measurements), and dashed lines denote 95% limits of agreement (1.96SD of the difference). (a) Overall (bias: −2.024, 95% LOA: −11.87 to 7.83). (b) Male (bias: −1.418, 95% LOA: −10.03 to 7.20). (c) Female (bias: −2.460, 95% LOA: −13.04 to 8.12).

Table 2 shows Spearman *r*, ICC, and Bland–Altman analysis results for the values of WC measured by two methods: manual and BIA. The Spearman *r* was also illustrated in Figure 1, and the Bland–Altman plots are shown in Figure 2. Overall, and in males and females, WC measured by BIA showed a very strong correlation with WC measured manually based on the Spearman *r* and almost perfect agreement based on ICC. The Bland–Altman analysis showed that bias (BIA-manual) was negative overall (−2.024), for males (−1.418) and for females (−2.460), suggesting an underestimation of the BIA compared with the manual methods. We also found that the difference was larger in females than in males. We found that the agreement between the WC measurements using BIA and manual methods were more consistent in males than in females. Interchangeability was found between the WC measured by BIA and the manual method because the percentage error was less than 15% overall (12.3%), for males (10.2%) and for females (13.8%).

Table 3 shows the comparisons between the normal group and the LS risk group according to sex. For overall participants, a significant difference was observed in terms of age (*P* < 0.001), sex (*P*=0.010), BMI (*P* < 0.001), PBF (*P* < 0.001), WC by BIA (*P*=0.003), and triglycerides (*P*=0.019) between the groups. For males, there were significant difference in terms of age (*P*=0.002), BMI (*P*=0.041), PBF (*P*=0.003), and WC by BIA (*P*=0.027); for females, there were significant differences in terms of age (*P*=0.007), weight (*P* < 0.001), BMI (*P* < 0.001), PBF (), aSMI (*P*=0.006), WC by BIA (*P* < 0.001), and triglycerides (*P*=0.006).

Table 4 shows the result of the logistic regression model for LS risk in all participants. WC by BIA (*P* < 0.001), age (*P* < 0.001), female (*P* < 0.001), and total cholesterol (*P*=0.016) were significantly associated with LS risk.

Table 5 shows the results of the logistic regression model according to sex. Only WC by BIA and age were significantly associated with LS risk in both males and females. From these results, it was shown that the increase in WC measurement by BIA significantly correlated LS risk as well as increase in age.

## 4. Discussion

WC is an obesity-related parameter that has been reported to be associated with various diseases and conditions [26–28]. Currently, WC measurement is performed manually. In large-scale health checkups, manual measurements of many participants within a limited time may cause interrater error because of increases in the number of measurers. To solve such problems, there is a need for a device that has a high degree of agreement with manual measurement and can be used to perform automatic measurements in a short period time. In recent years, BIA, a portable device with no exposure, has been used for measurement of body composition and anthropometric markers. We also use it in the context of health checkups. Because BIA can obtain a large amount of data in a short period of time using one measurement, it has the great advantages of reducing labor, time, and interrater errors compared to those associated with manual measurement. Advances in technology have also generated BIA devices that can automatically measure trunk circumferences including WC. Nevertheless, there has been no report on the validation of BIA with respect to manual measurement values. The present study was the first to validate WC measured by these two measurement methods in a large-scale prospective health checkup population. Furthermore, the Inbody 770 BIA device has been used to measure PBF and aSMI in various studies [29, 30], so it was thought that additional evidence could be accumulated.

Because manual WC measurement introduces errors, it is necessary to evaluate the agreement with the manual method when evaluating the accuracy of WC measurement using BIA. If the agreement is high, it is possible that the measurement modalities are interchangeable. Generally, in order to validate measured values using two measurement methods, Spearman correlation and ICC are used. However, correlation analysis is a method of evaluating the relationship between two different events, and the value of the correlation coefficient cannot evaluate differences or variations between measured values. Therefore, it is difficult to evaluate interchangeability only using these assessments. It is also necessary to evaluate the agreement using Bland–Altman analysis, a method of measuring by two methods and examining the differences between the measured values. In this study, the interchangeability of BIA and manual measurements was also evaluated based on past reports [23]. Then, for males and females, there was a high correlation between WC measured using BIA and manual methods, demonstrating interchangeability. This result is very important, and there is the possibility for future research can focus on the use of BIA not only for WC but also other trunk circumferences.

Moreover, because we confirmed that WC measurement by BIA is interchangeable with manual measurement, our previous report [6] was further developed and examined. In that report, we did not evaluate males and LS risk was not considered. The present study differs from our previous study in the following ways: we measure WC using BIA as well as the manual method; we increase the number of subjects, including males; and we include total cholesterol and triglyceride, as well as the evaluation of LS risk which is an indicator of mobility. With respect to the presence or absence of LS risk, the aim was to include an earlier stage; in the past, relevant factors have been investigated in various approaches in the same way [4, 5]. We found in univariate analysis that WC measurement by BIA was significantly high in the LS risk group overall, in males and in females. Furthermore, even if statistical adjustments were made for other factors in logistic regression analysis, the increase in WC measured by BIA was found to be a significant LS risk factor. This study has allowed us to further develop the previous report and to accumulate further evidence. It is important to continue focusing on the increase of WC and to improve early detection of LS risk.

This study has several limitations. First, it was a single-center study and, therefore, may be subject to selection bias. Multicenter studies are needed to validate our findings. Second, measurement differences may occur if BIA devices from different manufacturers are used. Therefore, standardization of technology and cross calibration of electrical resistance should be addressed in the future.

## 5. Conclusions

We found that WC measured by BIA (Inbody 770) is interchangeable with manual measurements. We also showed that even with statistical corrections for various relevant factors, the increases in WC measured by BIA were significantly associated with LS risk not only in females but also in males. The progress of BIA technology is remarkable; depending on the device, it is possible to measure trunk circumferences other than WC, and a large amount of data can be obtained in a short time using one measurement. Therefore, according to the results of this study, it may be possible to investigate on a large scale the relationship between trunk circumferences including WC and various diseases and conditions.

## Figures and Tables

**Table 1:** Demographic, anthropometric, blood test, and LS risk prevalence data of the study participants.

Variables	Total (*N* = 597)	Male (*N* = 250)	Female (*N* = 347)	*P* value
Age (years)	64.6 (10.1)	66.5 (9.3)	63.3 (10.4)	**<0.001**
Height (cm)	158.1 (8.5)	165.0 (6.1)	153.1 (6.2)	**<0.001**
Weight (kg)	59.2 (11.6)	66.9 (10.7)	53.7 (8.8)	**<0.001**
BMI (kg/m^2^)	23.6 (3.5)	24.5 (3.3)	22.9 (3.5)	**<0.001**
PBF (%)	29.1 (6.9)	25.2 (5.7)	31.9 (6.3)	**<0.001**
aSMI (kg/m^2^)	6.7 (1.0)	7.6 (0.8)	6.1 (0.7)	**<0.001**
WC by manual (cm)	81.8 (10.1)	86.3 (8.9)	78.5 (9.6)	**<0.001**
WC by BIA (cm)	79.8 (9.6)	84.9 (9.5)	76.1 (7.9)	**<0.001**
Total cholesterol (mg/dL)	208.6 (32.7)	203.6 (33.6)	212.1 (31.6)	**0.009**
Triglycerides (mg/dL)	107.3 (65.4)	122.4 (82.4)	96.5 (46.9)	**<0.001**
Prevalence of LS risk (%)	54.6%	48.4%	59.1%	**0.010**

Evaluated using the Mann–Whitney *U* test, Fisher's exact test. Parameter values are shown as means (standard deviations) or numbers. Bold values indicate a significant difference. PBF and aSMI were measured using Inbody 770 BIA unit. BMI, body mass index; PBF, percent body fat; WC, waist circumference; BIA, bioelectrical impedance analysis; aSMI, appendicular skeletal muscle index; LS, locomotive syndrome.

**Table 2:** Correlation coefficient (*r*), ICC, and Bland–Altman analysis in WC measured by two methods: manual and BIA.

	Total	Male	Female
Spearman *r*	0.871^*∗∗∗*^	0.875^*∗∗∗*^	0.830^*∗∗∗*^
ICC	0.930	0.940	0.896
Bland–Altman analysis			
Bias (BIA-manual)	−2.024	−1.418	−2.460
SD	5.025	4.395	5.398
95% LOA	−11.87 to 7.83	−10.03 to 7.20	−13.04 to 8.12
Percentage error (%)	12.3	10.2	13.8

^*∗∗∗*^ *P* < 0.001. *r*, correlation coefficient; ICC, interclass correlation coefficients; WC, waist circumference; BIA, bioelectrical impedance analysis; SD, standard deviation; LOA, limits of agreement.

**Table 3:** Comparison between the normal group and LS risk group according to sex.

Variables	Total			Male			Female		
	Normal (*N* = 271)	LS risk (*N* = 326)	*P* value	Normal (*N* = 129)	LS risk (*N* = 121)	*P* value	Normal (*N* = 142)	LS risk (*N* = 205)	*P* value
Age (years)	63.0 (9.8)	66.0 (10.1)	**<0.001**	64.6 (9.1)	68.5 (9.2)	**0.002**	61.5 (10.3)	64.6 (10.3)	**0.007**
Sex (male/female)	129/142	121/205	**0.010**						
Height (cm)	158.6 (8.4)	157.7 (8.5)	0.22	165.0 (6.0)	165.0 (6.1)	0.99	152.7 (5.6)	153.4 (6.5)	0.51
Weight (kg)	58.3 (11.2)	59.9 (11.9)	0.066	66.1 (9.5)	67.7 (11.9)	0.22	51.3 (7.3)	55.4 (9.3)	**<0.001**
BMI (kg/m^2^)	23.1 (3.1)	24.0 (3.8)	**<0.001**	24.2 (2.8)	24.8 (3.8)	**0.041**	22.0 (3.0)	23.6 (3.7)	**<0.001**
PBF (%)	27.5 (6.5)	30.5 (7.0)	**<0.001**	24.2 (4.9)	26.2 (6.2)	**0.003**	30.5 (6.2)	33.0 (6.3)	**<0.001**
aSMI (kg/m^2^)	6.8 (1.1)	6.7 (1.0)	0.46	7.6 (0.7)	7.5 (0.8)	0.31	6.0 (0.6)	6.2 (0.8)	**0.006**
WC by BIA (cm)	78.5 (8.9)	83.3 (10.7)	**0.003**	83.6 (7.9)	86.3 (10.8)	**0.027**	73.8 (6.8)	77.7 (8.2)	**<0.001**
Total cholesterol (mg/dL)	211.3 (34.7)	206.3 (30.9)	0.11	206.5 (35.8)	200.6 (31.0)	0.15	215.6 (33.1)	209.7 (30.4)	0.22
Triglycerides (mg/dL)	101.0 (57.8)	112.6 (70.7)	**0.019**	115.1 (68.7)	130.1 (94.5)	0.25	88.2 (42.0)	102.3 (49.4)	**0.006**

Evaluated using the Mann–Whitney *U* test and Fisher's exact test. Parameter values are shown as means (standard deviations). Bold values indicate significant difference. LS, locomotive syndrome; BMI, body mass index; PBF, percent body fat; WC, waist circumference; BIA, bioelectrical impedance analysis; aSMI, appendicular skeletal muscle index.

**Table 4:** Logistic regression model for LS risk in all the participants.

Variables	*β*	Odds ratio (95% CI)	*P* value
WC by BIA (cm)	0.102	1.108 (1.057–1.161)	**<0.001**
Age (years)	0.051	1.053 (1.033–1.073)	**<0.001**
Sex (male)	−1.420	0.242 (0.151–0.386)	**<0.001**
Total cholesterol (mg/dL)	−0.007	0.994 (0.988–0.999)	**0.016**
BMI (kg/m^2^)			0.051
Triglycerides (mg/dL)			0.20
PBF (%)			0.88

All variables (*P* < 0.25) that showed a certain degree of difference in univariate analysis were used as covariates. The dependent variable was LS risk. Covariates were age, sex, BMI, PBF, WC by BIA, total cholesterol, and triglycerides. Bold values type indicate significant difference. LS, locomotive syndrome; *β*, partial regression coefficient; CI, confidence intervals; WC, waist circumference; BIA, bioelectrical impedance analysis; BMI, body mass index; PBF, percent body fat.

**Table 5:** Logistic regression model for LS risk according to sex.

Male			*P* value	Female			*P* value
Variables	*β*	Odds ratio (95% CI)		Variables	*β*	Odds ratio (95% CI)	
WC by BIA (cm)	0.095	1.100 (1.029–1.176)	**0.005**	WC by BIA (cm)	0.134	1.143 (1.073–1.217)	**<0.001**
Age (years)	0.058	1.060 (1.027–1.093)	**<0.001**	Age (years)	0.051	1.052 (1.027–1.079)	**<0.001**
BMI (kg/m^2^)			0.080	Total cholesterol (mg/dL)			0.065
Total cholesterol (mg/dL)			0.24	PBF (%)			0.067
PBF (%)			0.48	Triglycerides (mg/dL)			0.20
Triglycerides (mg/dL)			0.52	aSMI (kg/m^2^)			0.25
				BMI (kg/m^2^)			0.44

All variables (*P* < 0.25) that showed a certain degree of difference in univariate analysis were used as covariates. The dependent variable was LS risk. Covariates in males were age, BMI, PBF, WC by BIA, total cholesterol, and triglycerides. Covariates in females were age, BMI, PBF, aSMI, WC by BIA, total cholesterol, and triglycerides. Bold values indicate significant difference. *β*, partial regression coefficient; LS, locomotive syndrome; CI, confidence intervals; WC, waist circumference; BIA, bioelectrical impedance analysis; BMI, body mass index; PBF, percent body fat; aSMI, appendicular skeletal muscle index.

## Data Availability

The data used to support the findings of this study are included within the article.
